# Inhibition of 2-Oxoglutarate Dehydrogenase as a Chemical Model of Acute Hypobaric Hypoxia

**DOI:** 10.3389/fmed.2021.751639

**Published:** 2021-12-17

**Authors:** Anastasia Graf, Alexander Ksenofontov, Victoria Bunik

**Affiliations:** ^1^Faculty of Biology, Lomonosov Moscow State University, Moscow, Russia; ^2^Faculty of Nano-, Bio-, Informational and Cognitive and Socio-Humanistic Sciences and Technologies, Moscow Institute of Physics and Technology, Moscow, Russia; ^3^Andrey Nikolaevich (A. N.) Belozersky Institute of Physicochemical Biology, Lomonosov Moscow State University, Moscow, Russia; ^4^Faculty of Bioengineering and Bioinformatics, Lomonosov Moscow State University, Moscow, Russia; ^5^Biochemistry Department, Sechenov University, Moscow, Russia

**Keywords:** brain metabolism of amino acids, hypoxia, 2-oxoglutarate dehydrogenase, pregnancy, succinyl phosphonate

## Abstract

Both hypoxia and inhibition of 2-oxoglutarate dehydrogenase complex (OGDHC) are known to change cellular amino acid pools, but the quantitative comparison of the metabolic and physiological outcomes has not been done. We hypothesize that OGDHC inhibition models metabolic changes caused by hypoxia, as both perturb the respiratory chain function, limiting either the NADH (OGDHC inhibition) or oxygen (hypoxia) supply. In the current study, we quantify the changes in the amino acid metabolism after OGDHC inhibition in the highly sensitive to hypoxia cerebellum and compare them to the earlier characterized changes after acute hypobaric hypoxia. In addition, the associated physiological effects are characterized and compared. A specific OGDHC inhibitor succinyl phosphonate (SP) is shown to act similar to hypoxia, increasing levels of many amino acids in the cerebellum of non-pregnant rats, without affecting those in the pregnant rats. Compared with hypoxia, stronger effects of SP in non-pregnant rats are observed on the levels of cerebellar amino acids, electrocardiography (ECG), and freezing time. In pregnant rats, hypoxia affects ECG and behavior more than SP, although none of the stressors significantly change the levels of cerebellar amino acids. The biochemical differences underlying the different physiological actions of SP and hypoxia are revealed by correlation analysis of the studied parameters. The negative correlations of cerebellar amino acids with OGDHC and/or tryptophan, shown to arise after the action of SP and hypoxia, discriminate the overall metabolic action of the stressors. More negative correlations are induced in the non-pregnant rats by hypoxia, and in the pregnant rats by SP. Thus, our findings indicate that the OGDHC inhibition mimics the action of acute hypobaric hypoxia on the cerebellar amino acid levels, but a better prediction of the physiological outcomes requires assessment of integral network changes, such as increases in the negative correlations among the amino acids, OGDHC, and/or tryptophan.

## Introduction

Molecular mechanisms of complex pathophysiological phenomena are often studied in their chemical models. For instance, pentylenetetrazol is widely employed to model epilepsy (1), while mitochondrial impairment in neurodegenerative diseases is often modeled by exposure of animals to inhibitors of the respiratory chain (2, 3). The high sensitivity of mitochondrial complex II to inhibition by malonate and 3-nitropropionic acid (3-NP) is used to model Huntington's disease (2, 4). Bioactivation of 1-methyl-4-phenyl-1,2,3,6-tetrahydropyridine (MPTP) to its toxic 1-methyl-4-phenylpyridinium cation by glial monoamine oxidase B (5) may contribute to Parkinson's-disease-like localization of the damage, making this inhibitor of complex I useful to create the disease models (6). However, interpretations of similar models employing another such inhibitor, rotenone, are complicated by its side effects, not considered in the original model suggestion. As revealed by now, some of the rotenone actions may be caused not only by targeting complex I of the respiratory chain (3, 7). Thus, translational value of the results obtained in the chemical models of pathologies essentially depends on the knowledge of molecular features of the complex pathophysiology, which are reproduced by the model, and those which characterize the substance-specific action. In particular, although mitochondrial impairment occurs upon inhibition of either complex I or II, different downstream pathways are activated by each of specific mitochondrial inhibitors, potentially reproducing the symptoms specific for particular neurodegenerative diseases (8).

Hypoxic damage to cells, tissues, and organisms is widely studied because hypoxia is a component of many different pathologies. The higher the oxygen dependence of a tissue or an organism, the higher their sensitivity to hypoxia and the more deleterious the consequences. For instance, as a major oxygen consumer, the brain is prone to increased risk of hypoxia-induced insults. Many diseases of the central nervous system (CNS), such as stroke, encephalopathies, and Parkinson's or Alzheimer's diseases, are associated with hypoxia. On the other hand, pregnancy imposes additional oxygen demands to support the mother and growing fetus, which is manifested in a higher sensitivity to hypoxia of pregnant vs. non-pregnant women (9). Hypoxia during pregnancy has profound adverse effects on maternal and fetal health, being the main cause of pregnancy complications, such as preeclampsia and intrauterine growth restriction. A challenging question is to what extent these specific physiological features are addressed by this or that model of hypoxia. However, very often, this question is ignored, whereas the usage of specific chemical models of hypoxia is hidden under the definition “hypoxic stress” (10, 11). For instance, a rather high concentration of CoCl_2_ (0.15 mM) is used to model hypoxia (11), although it is known that, despite the similarity in some changes induced by hypoxia or CoCl_2_ administration, their causes are disparate (12). Hypoxia perturbs the oxygen-requiring terminal step of the respiratory chain, i.e., the reaction catalyzed by cytochrome oxidase (complex IV of the respiratory chain). This perturbation decreases the mitochondrial capacity to produce energy through oxidative phosphorylation. The question arises, how similar the metabolic changes induced by hypoxia are to those induced by other perturbations of mitochondrial energy production? In a wider context, the question applies to the treatment of diseases having convergent symptoms yet induced by different molecular causes. We and others have shown that hypoxic damage to an organism includes perturbations in the amino acid pools (9, 13). Remarkably, this action of hypoxia is not reproduced in the CoCl_2_ models of hypoxia (12) but is highly reminiscent of the perturbations in the amino acid profiles by specific inhibition of the mitochondrial 2-oxoglutarate dehydrogenase multienzyme complex (OGDHC), observed in different biosystems *in situ* and *in vivo* (13). Apart from its involvement in amino acid metabolism, the OGDHC inhibition decreases the production of NADH to be oxidized by the respiratory chain. We, therefore, hypothesized that Succinyl phosphonate (SP) administration may be a fruitful model of hypoxia, reproducing not only the energetic impairments but also the metabolic action of hypoxia on the amino acid pool. As free amino acids are involved in protein synthesis, energy production, neurotransmission, and immunity, changes in cellular amino acid pools may be important mediators of the pathological cascades induced by hypoxia.

The goal of the current study is to characterize the inhibition of the Tricarboxylic Acid (TCA) cycle flux through OGDHC as a chemical model of hypoxia. To achieve this goal, we quantify the changes in the amino acid metabolism, induced by a short-term inhibition of OGDHC, and compare them to those after acute hypobaric hypoxia, characterized in our previous study (9). We reveal qualitatively similar, physiological-state-dependent consequences of the OGDHC inhibition and hypoxia for the levels of free amino acids. Using a previously developed sensitive approach to quantify metabolic changes beyond the steady-state concentration of metabolites (9, 14, 15), we show that the induced homeostatic responses, manifested in the insult-specific sets of changed metabolic correlations, are not necessarily the same for SP and hypoxia. As a result, the biochemical actions of SP and hypoxia are more similar in the non-pregnant than pregnant rats. The characterized physiological effects of OGDHC inhibition and hypoxia are shown to depend not only on the changed levels of amino acids but also on the induction of negative interdependences between amino acids and OGDHC and/or tryptophan by SP or hypoxia.

## Materials and Methods

### Animals

All the experiments were in accordance with the Guide for the Care and Use of Laboratory Animals (European Union Directives 86/609/EEC and 2010/63/EU), and approved by the Bioethics Committee of Lomonosov Moscow State University (protocol number 69-o from 09.06.2016). The animals were kept at 21 ± 2°C and relative humidity 53 ± 5% with the 12/12 h light/dark cycle (lights on 9:00 and lights off 21:00). The rats were purchased from the State Research Center of the Russian Federation—Institute for Biomedical Problems, Russian Academy of Sciences, and were adapted to our conditions for 2 weeks before they entered experimental settings. Five to six animals were kept in one T/4K cage. Standard rodent pellet food (laboratorkorm.ru) and tap water were available *ad libitum*.

Wistar female rats, pregnant (*n* = 29) and non-pregnant (*n* = 38), of about 250–300 g (2.5–3.0 months) were used in the experiment. Two virgin female rats were located in a cage with one male. After 24 h, vaginal smears were analyzed. The first day of pregnancy was considered to be the day of the sperm detection in the vaginal smear. After that, the male rats were removed from the cage.

Six groups of female rats were used for comparison: (1) normoxic non-pregnant, *n* = 17; (2) hypoxic non-pregnant, *n* = 8; (3) non-pregnant with intranasal administration of SP, *n* = 13; (4) normoxic pregnant, *n* = 8; (5) hypoxic pregnant, *n* = 10; and (6) pregnant with intranasal application SP, *n* = 11. The animals from several independent experiments were pooled. The animals were decapitated after the physiological assessment was completed, 24 h after the treatments. Cerebella were quickly excised and stored at −70°C prior to the biochemical analyses. A half of each cerebellum was used to prepare the homogenates for enzymatic assays, the other half—to prepare the methanol-acetic acid extracts for the amino acid analysis.

### Acute Hypobaric Hypoxia

Female rats were exposed to hypobaric hypoxia in a decompression (altitude) chamber by decreasing the atmospheric pressure, as described previously (9). The pregnant rats were exposed to hypoxia at the 9–10th day of pregnancy, roughly corresponding to the first trimester of human pregnancy (16, 17). This period is critical for organogenesis.

### Administration of the OGDH Inhibitor SP to Animals

The animals received SP at 5 mg/kg by intranasal application of the water solution of the trisodium salt, with 0.9% NaCl substituting for SP in all reference groups. Intranasal administration was applied as it provided high bioavailability of the administered substances to the brain (18, 19). Administration of SP to pregnant rats was done on the 9–10th day of pregnancy.

### Behavioral Parameters

The “Open Field” test (“OpenScience”, Moscow, Russia) was used to quantify anxiety level, exploratory, and locomotor activities 24 h after exposure to SP or hypoxia. Exploratory activity and anxiety were assessed cumulatively as described previously (20).

### Electrocardiography

Electrocardiography (ECG) was registered as described previously (20) using non-invasive ECG recording. The balance of autonomous regulation of heart rate was assessed according to the published method (21) by calculating the following parameters: average R–R interval in a sample, ms; parasympathetic, or relaxation, index of the state of the nervous system—RMSSD; sympathetic, or stress, index of the state of the nervous system—SI (21, 22).

### OGDHC Assays

We used euthanasia by decapitation 24 h after a treatment (0.9% sodium chloride, SP, or hypoxia). The cerebella were quickly excised on ice, frozen in liquid nitrogen, and stored at −70°C. Homogenization of cerebella and assays of their OGDHC activity were performed as described earlier (23) with the recent modifications introduced in (22).

### Ninhydrine Quantification of Amino Acids

Methanol/acetic acid extracts of cerebella were prepared according to a published protocol (24) and stored at −70°C. Amino acids were quantified in 50 μl of the extracts injected to an ion-exchange column 2622SC(PH) (Hitachi, Ltd., P/N 855-3508, 4.6 ×80 mm, Japan), eluted by the step gradient of sodium-acetate buffers according to the published procedure (9).

### Data Acquisition and Statistics

Statistical analysis was performed using the GraphPad Prism 7.0 software (GraphPad Software Inc., CA, USA) and Statistica 10.0 (StatSoft Inc., Tulsa, OK, USA). The values are presented as means ± SEM. Statistical significance of differences upon the comparison of more than the two experimental groups was assessed using two-way ANOVA with Tukey's *post-hoc* test. The Pearson′s correlations between the levels of different amino acids or between the levels of amino acids and OGDHC activity were characterized by the correlation coefficients and *p-*values of the correlation. Statistical significance of differences in the elaborated earlier parameters to characterize metabolic interactions between the levels of OGDHC activity and/or amino acids (9, 14, 15), was assessed by the Wilcoxon signed-rank test. Differences with *p* ≤ 0.05 were considered significant. Differences with 0.05 < *p* ≤ 0.1 were considered as trends.

## Results

### Changes in the Levels of Free Amino Acids in Cerebella of the SP-Exposed vs. Control Female Rats Depend on Pregnancy, Similar to the Changes After Acute Hypobaric Hypoxia

**Figure 1:**
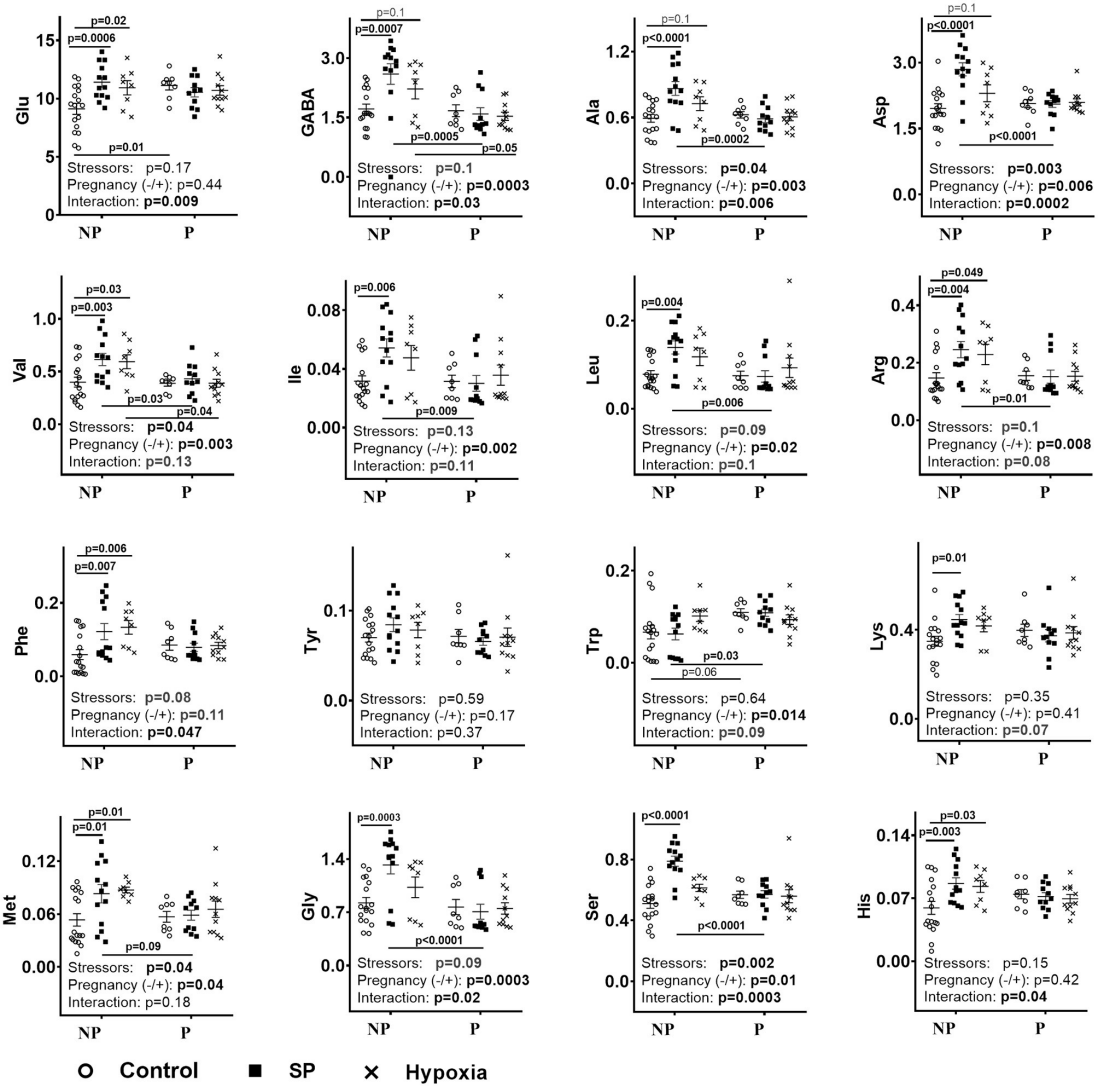
Influence of succinyl phosphonate (SP) on the cerebellar levels of amino acids in the non-pregnant (NP) and pregnant (P) rats and comparison of the SP effects to those of acute hypobaric hypoxia. The content of free amino acids indicated on *Y*-axes is shown in micromoles per grams of fresh tissue weight. NP—non-pregnant rats: control *n* = 17, SP *n* = 13, hypoxia *n* = 8. P—pregnant rats: control *n* = 8, SP *n* = 11, hypoxia *n* = 10. The comparison of the effects of SP and hypoxia employs the data on hypoxic rats obtained earlier (9). Statistical analysis by two-way ANOVA is described in Methods. Statistically significant differences between the groups determined by the *post-hoc* test, and significance of the factors (stressors SP or hypoxia and pregnancy) and their interaction determined by ANOVA, are given on the graphs.

Figure 1 (NP) shows that exposures of non-pregnant female rats to either SP or acute hypobaric hypoxia have similar effects, increasing the levels of many amino acids in the cerebellum. In most cases, the levels of the amino acids are increased more by SP than hypoxia. This feature is evident by comparison of not only the corresponding mean values but also the values of p characterizing the statistical significance of the differences to the control groups.

As shown earlier, pregnant rats mostly do not differ from non-pregnant rats in their cerebellar amino acid levels, with only glutamate and tryptophan being higher in the pregnant vs. non-pregnant rats (9). However, neither hypoxia nor SP changes the cerebellar amino acids in pregnant rats (Figure 1, P). Due to these disparate effects on the non-pregnant and pregnant rats, both SP and hypoxia induce differences between these rat groups in cerebellar levels of many amino acids, which are absent before the treatments. Regarding the pre-existing differences, both treatments eliminate the difference in the glutamate levels, whereas the difference in the tryptophan level is preserved after SP treatment, but lost after hypoxia (Figure 1, NP vs. P).

### Correlation Analysis of Changes in Metabolic Interdependence Between the Cerebellar OGDHC Activity and/or Amino Acids Levels in the Female Rats

Correlation analysis characterizing the relationships between the quantified parameters is a powerful tool to unravel integral changes in metabolic networks, which may be hidden upon analysis of the mean values because of homeostatic mechanisms, effected to stabilize these values (9, 14, 15). The correlations of Supplementary Table 1 form the condition- and physiological-state-dependent patterns which may be quantified by the summarized and mean correlation coefficients, and the number of positive and negative correlations, presented in Table 1. This analysis reveals a significant difference in the correlation patterns of the non-pregnant and pregnant rats (Table 1, Supplementary Table 1), reciprocating the different reactivity of the amino acid levels in these rats to SP exposure (Figure 1). SP induces negative correlations between the OGDHC activity and several amino acids in the non-pregnant rats (Supplementary Table 1A), with the effect not pronounced in the pregnant rats (Supplementary Table 1B). Instead, in the cerebella of pregnant rats, SP induces negative correlations between tryptophan and several amino acids (Supplementary Table 1B). This metabolic action is specific to SP, as it is not observed in the pregnant rats exposed to hypoxia (9). The summarized correlations show the degree of interdependence between the components of the metabolic network, characterizing the network state (9, 15). In particular, the sum and average of the correlation coefficients tend to increase interdependence in the SP-treated non-pregnant rats vs. the control group (*p* ~ 0.1), but only the difference in the number of negative correlations in these groups is statistically significant (*p* = 0.018) (Table 1). In contrast, in pregnant rats, SP strongly increases all the parameters characterizing the interdependence between OGDHC activity and/or amino acids levels (Table 1). A higher increase in interdependence of amino acids in the SP-exposed pregnant vs. non-pregnant rats, compared with the respective control groups, manifests greater reorganization of the metabolic network in response to SP in the pregnant vs. non-pregnant rats. This is in good accord with the higher stabilization of the amino acid levels in the cerebella of the pregnant vs. non-pregnant rats (Figure 1).

**Table 1:** Analysis of the succinyl phosphonate SP-induced changes in interdependence of the levels of amino acids and OGDHC activity in cerebella of the NP and P rats.

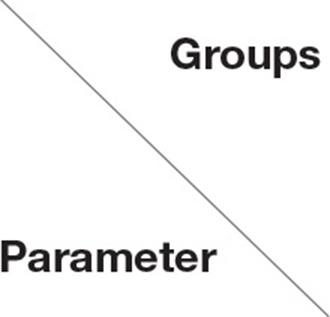
	**Non-pregnant rats (NP)**								**Pregnant rats (P)**							
		**∑**		**X**		**+**		**–**		**∑**		**X**		**+**		**–**	
	**Control**	**SP**	**Control**	**SP**	**Control**	**SP**	**Control**	**SP**	**Control**	**SP**	**Control**	**SP**	**Control**	**SP**	**Control**	**SP**
OGDHC	4.27	6.96	0.27	0.43	1	0	0	6	6.04	5.08	0.38	0.32	0	0	0	1
ALA	9.45	11.78	0.59	0.74	10	12	0	1	8.95	11.06	0.60	0.69	3	9	0	0
ARG	10.89	11.73	0.68	0.73	13	12	0	0	10.19	11.47	0.68	0.72	5	11	0	1
ASP	8.65	8.99	0.54	0.56	9	8	0	1	9.12	6.97	0.61	0.44	0	2	0	1
GABA	10.21	9.93	0.64	0.62	11	10	0	1	9.29	11.70	0.62	0.73	5	11	0	1
GLU	10.59	10.40	0.66	0.65	14	11	0	1	5.28	4.89	0.35	0.31	0	1	0	1
GLY	9.62	11.25	0.60	0.70	10	11	0	0	9.28	11.59	0.62	0.72	4	10	0	1
HIS	9.61	11.62	0.60	0.73	11	12	0	0	5.82	9.44	0.39	0.59	1	7	0	0
ILE	10.59	11.69	0.66	0.73	12	12	0	1	9.81	11.68	0.65	0.73	6	11	0	1
LEU	10.39	11.58	0.65	0.72	12	12	0	1	9.95	11.70	0.66	0.73	6	11	0	1
LYS	7.24	10.81	0.45	0.68	4	11	0	0	9.72	10.27	0.65	0.64	2	8	0	1
MET	8.42	6.29	0.53	0.39	9	5	0	0	5.67	9.97	0.38	0.62	0	9	0	2
PHE	9.89	7.84	0.62	0.49	8	8	0	0	9.21	11.70	0.61	0.73	1	12	0	1
SER	8.16	8.76	0.51	0.55	12	9	0	0	7.78	8.06	0.52	0.50	1	3	0	1
TRP	4.49	5.97	0.28	0.37	2	3	0	0	6.13	10.32	0.41	0.65	0	0	0	10
TYR	9.18	11.77	0.57	0.74	11	13	0	0	10.49	9.05	0.70	0.57	5	8	0	0
VAL	10.63	6.92	0.66	0.43	13	7	0	0	5.09	9.70	0.34	0.61	1	7	0	1
Sum or Average	**152.28**	**164.27**	**0.56**	**0.60**	**162**	**156**	**0**	**12**	**137.82**	**164.67**	**0.54**	**0.61**	**40**	**120**	**0**	**24**
*p* vs. control	*0.11*		*0.12*				**0.018**		**0.05**		**0.04**		**0.0007**		**0.0014**	
*p* NP vs. P													**0.05**	**0.0009**	*0.14*	

*For the 2-oxoglutarate dehydrogenase complex (OGDHC) activity (OGDHC) and each of the amino acids, the arithmetic sum of its correlation coefficients (absolute values) to other amino acids (Σ), average correlation coefficient (X) calculated from the absolute values, and the total number of statistically significant positive (+) and negative (–) correlations are shown. At the bottom, the sum (in case of Σ, positive and negative correlations) or average (in case of X) of all the values in the row are presented, along with p values of the differences between these parameters in the control and SP groups (p vs. control), or between NP and P rats (p NP vs. P), estimated by the Wilcoxon signed rank test. NP rats: control n = 17. SP n = 13; P rats: control n = 8. SP n = 11. The parameters exhibiting statistically significant differences and the corresponding p-values are marked in bold. The p-values at the level of a trend are shown in italic*.

Thus, the stabilization of the amino acid levels in the cerebellum of SP-treated pregnant rats (Figure 1, P) is achieved along with their increased interdependence (Table 1). In the non-pregnant rats, where the less prominent perturbation in the metabolic interdependence between OGDHC and/or amino acisd is observed in the SP-treated vs. control rats (Table 1), the levels of amino acids increase (Figure 1, NP).

### Comparison of the Changes in the Amino Acid Metabolism, Induced by SP or Hypoxia

The impact of SP on the metabolic interdependencies between OGDHC and/or amino acids in both the non-pregnant and pregnant rats is most robustly observed from the increased number of the negative correlations (Table 1 and Supplementary Table 1). However, this effect of SP involves different components in different metabolic networks. In the non-pregnant rats, it is the OGDHC activity that becomes negatively correlated with multiple amino acids, whereas in the pregnant rats it is the tryptophan level that becomes negatively correlated with many amino acids (Supplementary Table 1).

Thus, in addition to the levels of free amino acids (Figure 1) or arithmetic sums characterizing the metabolic interdependencies in general (i.e., both positive and negative ones) (9, 14, 15), the abundance of negative metabolic interdependencies of amino acids with the OGDHC activity or tryptophan levels is estimated in this work to compare the metabolic actions of SP and acute hypobaric hypoxia. Table 2 shows that both SP and hypoxia induce negative interdependencies. However, this change occurs to different degrees, dependent on specific perturbation (SP or hypoxia) or physiological state (pregnant or non-pregnant). In the non-pregnant rats, hypoxia increases negative interdependencies of both OGDHC and tryptophan, while SP does so with the OGDHC interdependencies only. Moreover, the algebraic sum of the correlation coefficients (Table 2) indicates that the increase in the negative interdependencies by SP is less pronounced than the one by hypoxia. Thus, in the non-pregnant rats, SP increases the negative correlations less than hypoxia does, while the opposite is observed in the pregnant rats (Table 2).

**Table 2:** Induction by SP or acute hypobaric hypoxia of negative correlations of OGDHC or tryptophan with amino acids in the cerebella of NP or P rats.

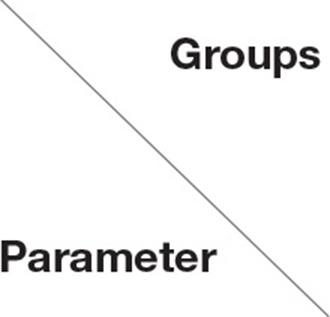
	**Non-pregnant rats (NP)**						**Pregnant rats (P)**					
		**∑** **OGDHC**			**∑** **TRP**			**∑** **OGDHC**			**∑** **TRP**		
	**Control**	**SP**	**Hypoxia**	**Control**	**SP**	**Hypoxia**	**Control**	**SP**	**Hypoxia**	**Control**	**SP**	**Hypoxia**
TRP or OGDHC	0.71	0.46	0.53	0.71	0.46	0.53	0.85	−0.32	0.05	0.85	−0.32	0.05
ALA	0.12	−0.58	−0.94	0.39	0.15	−0.68	−0.35	−0.34	−0.17	−0.21	−0.58	−0.02
ARG	−0.3	−0.45	−0.94	−0.14	0.25	−0.66	−0.33	−0.25	−0.03	−0.45	−0.77	−0.52
ASP	−0.47	−0.66	−0.9	−0.4	–*0.44*	−0.33	−0.04	−0.67	−0.06	0.01	−0.04	0.58
GABA	−0.4	−0.61	−0.95	−0.3	−0.32	−0.56	−0.55	−0.21	−0.12	−0.55	−0.73	−0.45
GLU	0.06	−0.72	−0.84	0.3	−0.36	−0.39	0.33	–*0.58*	−0.22	0.64	0.52	0.89
GLY	−0.4	−0.35	−0.95	−0.41	0.39	−0.56	−0.51	−0.23	−0.15	−0.59	−0.78	−0.49
HIS	−0.11	−0.39	−0.94	−0.05	0.40	−0.58	0.76	−0.32	0.03	0.53	−0.43	0.3
ILE	−0.35	−0.58	−0.95	−0.1	0.04	−0.56	−0.44	−0.21	0.14	−0.46	−0.8	−0.39
LEU	−0.31	−0.64	−0.95	−0.17	−0.04	−0.6	−0.42	−0.24	0.19	−0.48	−0.78	−0.27
LYS	0.28	−0.29	−0.69	0.62	0.39	−0.67	0.07	0.27	0.24	−0.08	−0.84	0.33
MET	0	0	−0.65	−0.05	0.77	−0.43	−0.15	0.24	0.63	−0.63	−0.91	−0.31
PHE	−0.24	−0.17	−0.95	−0.25	0.06	−0.6	−0.04	−0.11	0.08	−0.43	−0.81	−0.64
SER	0.03	−0.5	−0.19	0.01	−0.34	−0.11	0.36	0.42	0.44	0.02	−0.66	0.12
TYR	0.32	−0.45	−0.89	0.47	0.69	−0.63	−0.08	−0.32	0.13	−0.26	−0.57	0.09
VAL	−0.18	0.35	0.11	−0.11	0.024	0.69	0.76	0.48	0.36	0.79	0.22	−0.79
Sum	–**1.24**	–**5.58**	–**11.09**	**0.52**	**2.12**	–**6.14**	**0.22**	–**2.39**	**1.54**	–**1.3**	–**8.28**	–**1.52**
*p* vs. control	**0.008** **0.0008**				**0.005**					**0.0004**		
*p* SP vs. hypoxia	***0.002***			***0.01***			***0.001***			***0.006***		
*p* P vs. NP							**0.007** **0.0006**			**0.006** **0.003**		

*∑ OGDHC—the algebraic sum of correlation coefficients of the OGDHC activity and each of the amino acids*.

*∑ TRP—the algebraic sum of correlation coefficients of tryptophan and each of the other amino acids or OGDHC activity*.

*NP rats: control n = 17, SP n = 13, hypoxia n = 8. P rats: control n = 8, SP n = 11, hypoxia n = 10*.

*The three bottom lines show p values of the statistically significant (p < 0.05) differences between the indicated groups, estimated by the Wilcoxon signed rank test*.

*The strength of the negative correlations is estimated by the algebraic sum of correlation coefficients of OGDHC or tryptophan with other components. The algebraic sums for the hypoxic rats have been calculated using the published data (9). The parameters exhibiting statistically significant differences and the corresponding p-values are marked in bold. The p-values at the level of a trend are shown in italic*.

### Comparison of the Physiological Effects of SP and Hypoxia

**Figure 2:**
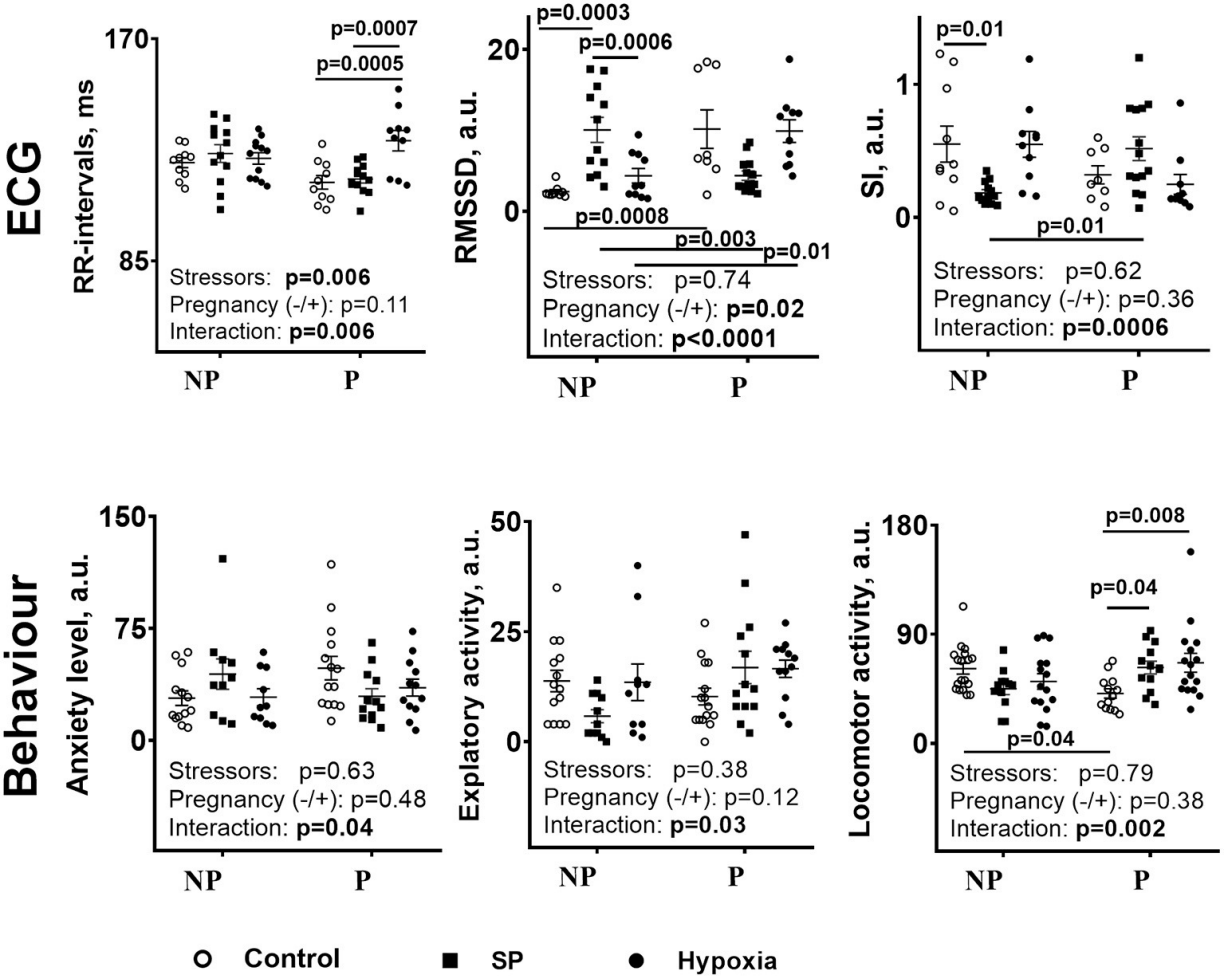
Comparison of the influence of SP or acute hypobaric hypoxia on physiological parameters of the NP and P rats. NP—the non-pregnant rats: control *n* = 17, SP *n* = 13, hypoxia *n* = 12. P—pregnant rats: control *n* = 10, SP *n* = 13, and hypoxia *n* = 10. Statistical analysis by two-way ANOVA is described in Methods. Both the *post-hoc*-test-determined statistically significant differences between the groups and ANOVA-determined significance of the factors (stressors SP or hypoxia and pregnancy) and their interaction are given on the graphs.

The data presented in Figure 2 compare the physiological actions of SP or hypoxia. First of all, similar to the biochemical parameters (Figure 1), the physiological ones (Figure 2) show different reactivities to SP and/or hypoxia in the non-pregnant and pregnant rats. The RMSSD and SI are changed by SP and/or hypoxia in the non-pregnant rats, whereas the R–R intervals, anxiety, and locomotion—in the pregnant rats. As a result, differences in the ECG and behavioral parameters between the non-pregnant and pregnant rats arise, (Figure 2, SI), disappear (Figure 2, locomotion, anxiety), or reverse (Figure 2, RMSSD), similar to the differences in the cerebellar amino acids content (Figure 1). Second, in the non-pregnant rats, a more pronounced, compared with hypoxia, the action of SP on animal physiology is seen in RMSSD and SI (Figure 2), similar to the more pronounced action of SP vs. hypoxia on the cerebellar amino acids content (Figure 1). In the pregnant rats, anxiety is decreased and locomotion is increased by SP, not hypoxia (Figure 2), in good accord with the SP-specific induction of the negative correlations of tryptophan with amino acids and OGDHC (Table 2). Finally, the different actions of SP and hypoxia on the tryptophan correlations in the pregnant rats (Table 2), associated with the disappearance of the difference in the tryptophan levels between the pregnant and non-pregnant rats after hypoxia (Figure 1), corresponds to the hypoxia-specific action on R–R interval in the pregnant rats (Figure 2, R–R interval).

Thus, the actions of SP and hypoxia on the non-pregnant and pregnant rats demonstrate several common features when the cerebellar amino acid content (Figure 1) and metabolic interactions (Table 2) are compared. The physiological responses (Figure 2) correspond to quantitative differences between the SP- and hypoxia-induced changes.

## Discussion

In this study, we have established a chemical model of acute hypoxia by showing high similarity in the actions of a specific inhibitor of OGDHC (SP) and acute hypobaric hypoxia on the cerebellar amino acid pool. The similarity is obvious from quantifications of the average levels of the cerebellar amino acids, and change reactivities of the amino acids to SP or hypoxia with pregnancy (Figure 1). Similar physiological consequences of animal exposure to acute hypobaric hypoxia or SP are also known from our previous studies, where the dependence of such responses on physiological differences imposed by sex has been shown upon exposure to SP or hypoxia of the adult animals (25) and in utero (26). From the metabolic viewpoint, the similarity is based on the fact that mitochondrial NADH oxidation by oxygen in the respiratory chain may be impaired both by inhibition of OGDHC and hypoxia. In the former case, the impairment is due to the limited NADH production in the tricarboxylic acid cycle, whereas in the latter case it is due to the limited oxygen availability. Remarkably, similarity in the action of the perturbed OGDHC function and hypoxia has been recently characterized by the stabilization of the hypoxia-inducible factor (HIF) in both cases (27). Thus, in good accord with the intimate relationship between metabolic perturbations and signaling systems whose action is always directed to normalize the perturbation, similar consequences of the OGDHC inhibition and hypoxia are revealed at both the metabolic and signaling levels.

More specific insights in the SP- or hypoxia-induced changes in the amino acid metabolic network are revealed by correlation analysis of the interdependencies between the network components (9, 15). In particular, this analysis pinpoints another common feature of the action of SP and hypoxia, which is the reversal of the correlation sign from positive to negative for many correlations of OGDHC or tryptophan with amino acids. The appearance of the negative correlations between OGDHC and amino acids may be interpreted as increased degradation of amino acids through OGDHC. The appearance of the negative correlations between amino acids and tryptophan, associated with preservation of the difference in the tryptophan levels between the non-pregnant and pregnant rats, implies that the levels of tryptophan upon the action of SP in pregnant rats are stabilized at the expense of other amino acids.

Compared with SP, hypoxia induces more negative metabolic interdependences in the non-pregnant rats (Table 2), associated with a higher stabilization of the levels of amino acids in the non-pregnant rats after hypoxia than after SP (Figure 1, NP). In contrast, in pregnant rats, hypoxia affects the interdependencies of either OGDHC or tryptophan much less than SP does, which is especially obvious regarding tryptophan (Table 2). As mentioned above, the SP-changed correlations of tryptophan in the pregnant rats presumably preserve the difference in the tryptophan levels between the pregnant and non-pregnant rats. Indeed, the difference is lost after hypoxia not affecting the tryptophan correlations in the pregnant rats (Table 2, Supplementary Table 1, Figure 1, Trp). It thus appears that the OGDHC- or tryptophan-involving pathways of the female rat cerebellum are the pathways with the highest reorganization during the perturbations induced by SP or hypoxia. Yet the reorganization of these pathways may occur to a different degree depending on physiological settings (pregnancy) and perturbation type (SP or hypoxia). These quantitative differences (Table 2) manifest in the stressor-specific physiological outcomes (Supplementary Table 1).

Supplementary Table 2 compares the overall changes in the amino acids/OGDHC metabolic network, induced by SP (characterized in the current study), and acute hypobaric hypoxia [studied previously in reference (9)]. The comparison indicates that the SP action is more similar to that of hypoxia in the non-pregnant than pregnant rats (Supplementary Table 2). Although increases in the summarized and average correlation coefficients are less pronounced after SP treatment (*p* = 0.1) compared with hypoxia (*p* < 0.01) (Supplementary Table 2A), the number of the negative correlations of the amino acid levels with the OGDHC activity is significantly increased by both SP and hypoxia (Supplementary Table 2B). This analysis confirms the leading role of the reorganization of the OGDHC-involving pathways in the SP- or hypoxia-induced perturbations. In the pregnant rats, the consequences of the actions of SP and hypoxia on the free amino acid levels are similar (Figure 1), yet this similarity is achieved through different adaptations of the metabolic network to the insults (Table 2 and Supplementary Table 2). The difference is due to the fact that SP strongly affects the tryptophan-involving pathways only in the pregnant rats, while hypoxia exerts such an effect in the non-pregnant rats. The finding that in the non-pregnant rats, hypoxia strongly affects both the OGDHC and tryptophan interdependencies with amino acids, corresponds to a higher impact of hypoxia vs. SP on the amino acid metabolic network in this animal group (Supplementary Table 2). In contrast, in the pregnant rats, it is the SP-induced perturbation in the tryptophan metabolic network, not observed after hypoxia, that provides for a higher network impact of SP vs. hypoxia (Supplementary Table 2). These quantitative differences between the SP and hypoxia network action in pregnant rats manifest in the physiological effects of SP on the anxiety and locomotor activity, absent in the hypoxic rats (Figure 2).

In view of the critical contribution of tryptophan metabolism to the cerebellar response to SP or hypoxia (Figure 1; Table 2), multiple studies pointing to the pregnancy-changed tryptophan metabolism are worth noting. This change is important both for fetus and mother, associated with the pregnancy-increased metabolic, energy, and oxygen demands (28–32). In pregnant rats, serotonin (33) and NAD (34) syntheses from tryptophan are increased, with the kinurenine pathway of the tryptophan degradation being critical for early embryonic brain development (35). The preservation by SP of the differences in the tryptophan levels in the pregnant vs. non-pregnant rats, which is not observed after hypoxia (Figure 1), may contribute to the SP-exerted protection from the action of hypoxia, known from our earlier studies of the effects of SP and/or hypoxia on adult animals and offspring (22, 23, 25, 26). The importance of amino-acids-related metabolism and signaling in pregnancy is underlined by the mTOR-controlled interaction between fetal growth and maternal supply (36). As discussed in our previous work (9), pregnancy-imposed changes in the mTOR signaling may be linked to the degradation of amino acids through the OGDHC-limited tricarboxylic acid cycle. This is supported by the critical difference in responses of the OGDHC-related amino acid metabolism to SP and hypoxia between the non-pregnant and pregnant rats, shown in our current work.

## Conclusions

Regarding the cerebellar metabolism of amino acids, specific inhibition of OGDHC mimics acute hypobaric hypoxia, with the similarity of the metabolic actions higher in the non-pregnant than pregnant rats. The highest impact of the OGDHC inhibition and hypoxia is shown on the pathways involving OGDHC and tryptophan.

## Data Availability Statement

The original contributions presented in the study are included in the article/Supplementary Material, further inquiries can be directed to the corresponding author/s.

## Ethics Statement

The animal study was reviewed and approved by the Ethics Committee of Lomonosov Moscow State University (protocol number 69-o from 09.06.2016).

## Author Contributions

VB: conceptualization, writing—original draft preparation, supervision, project administration, and funding acquisition. AG and AK: methodology, software, and validation. AG: formal analysis, investigation, data curation, and visualization. AG, AK, and VB: resources and writing—review and editing. All authors read and agreed to the published version of the manuscript.

## Funding

This research was funded by the Russian Science Foundation grant number N 18-14-00116 to VB.

## Conflict of Interest

The authors declare that the research was conducted in the absence of any commercial or financial relationships that could be construed as a potential conflict of interest.

## Publisher's Note

All claims expressed in this article are solely those of the authors and do not necessarily represent those of their affiliated organizations, or those of the publisher, the editors and the reviewers. Any product that may be evaluated in this article, or claim that may be made by its manufacturer, is not guaranteed or endorsed by the publisher.
