# Supplementary material for: Inhibition of 2-Oxoglutarate Dehydrogenase as a Chemical Model of Acute Hypobaric Hypoxia
Source: Front Med (Lausanne). 2021 Dec 17;8:751639. doi: 10.3389/fmed.2021.751639 (PMC8718613; doi:10.3389/fmed.2021.751639)
Supplement: Supplementary file 1 [file Data_Sheet_1.docx]

Supplementary Material

**Supplementary Table 1.** Correlation matrices characterizing interdependence between the amino acid levels and OGDHC activity in the cerebella of the non-pregnant (A) and pregnant (B) rats from the control (C) and SP -treated (SP) groups

| 1. **Non-Pregnant Rats** | | | | | | | | | | | | | | | | | | | | | | | | | | | | | | | | |  | |  |
| --- | --- | --- | --- | --- | --- | --- | --- | --- | --- | --- | --- | --- | --- | --- | --- | --- | --- | --- | --- | --- | --- | --- | --- | --- | --- | --- | --- | --- | --- | --- | --- | --- | --- | --- | --- |
| **SP**  **C** | **OGDHC** | | **ALA** | | **ARG** | | **ASP** | | **GABA** | | **GLU** | | **GLY** | | **HIS** | | **ILE** | | **LEU** | | **LYS** | | **MET** | | **PHE** | | **SER** | | **TRP** | | **TYR** | | **VAL** | |  |
| **OGDHC** |  | | **-0.58**  **0.038** | | *-0.45*  *.126* | | **-0.66**  **0.014** | | **-0.61**  **0.028** | | **-0.72**  **0.005** | | -0.35  0.234 | | -0.39  0.184 | | **-0.58**  **0.038** | | **-0.64**  **0.018** | | -0.29  0.344 | | 0.00  0.992 | | -0.17  0.574 | | *-0.50*  *0.081* | | *0.46*  *0.116* | | -0.45  0.128 | | 0.11  0.712 | |  |
| **ALA** | 0.12  0.670 | |  | | **0.93**  **0.000** | | **0.70**  **0.008** | | **0.81**  **0.001** | | **0.81**  **0.001** | | **0.91**  **0.000** | | **0.88**  **0.000** | | **0.95**  **0.000** | | **0.95**  **0.000** | | **0.82**  **0.001** | | *0.46*  *0.116* | | **0.79**  **0.001** | | **0.67**  **0.011** | | 0.15  .630 | | **0.89**  **0.000** | | *0.50*  *0.085* | |  |
| **ARG** | -0.30  0.273 | | **0.69**  **0.005** | |  | | **0.57**  **0.041** | | **0.68**  **0.010** | | **0.77**  **0.002** | | **0.89**  **0.000** | | **0.95**  **0.000** | | **0.95**  **0.000** | | **0.92**  **0.000** | | **0.92**  **0.000** | | *0.52*  *0.068* | | **0.87**  **0.000** | | **0.60**  **0.031** | | 0.25  .414 | | **0.96**  **0.000** | | *0.50*  *0.080* | |  |
| **ASP** | *-0.47*  *0.077* | | **0.57**  **0.028** | | **0.63**  **0.012** | |  | | **0.92**  **0.000** | | **0.90**  **0.000** | | *0.52*  *0.069* | | *0.51*  *0.075* | | **0.76**  **0.002** | | **0.81**  **0.001** | | *0.43*  *0.145* | | -0.08  0.806 | | 0.23  0.444 | | **0.81**  **0.001** | | *-0.44*  *0.129* | | **0.62**  **0.024** | | -0.03  .925 | |  |
| **GABA** | *-0.40*  *0.145* | | **0.68**  **0.005** | | **0.84**  **0.000** | | **0.85**  **0.000** | |  | | **0.90**  **0.000** | | **0.66**  **0.015** | | **0.59**  **0.034** | | **0.81**  **0.001** | | **0.85**  **0.000** | | *0.53*  *0.060* | | 0.07  .832 | | 0.39  .184 | | **0.86**  **0.000** | | -0.32  .282 | | **0.70**  **0.007** | | 0.24  .434 | |  |
| **GLU** | 0.06  0.845 | | **0.89**  **0.000** | | **0.77**  **0.001** | | **0.60**  **0.017** | | **0.67**  **0.006** | |  | | **0.65**  **0.017** | | **0.69**  **0.009** | | **0.90**  **0.000** | | **0.93**  **0.000** | | **0.62**  **0.025** | | 0.05  0.875 | | 0.41  0.159 | | **0.82**  **0.001** | | -0.36  0.225 | | **0.76**  **0.003** | | 0.13  0.675 | |  |
| **GLY** | *-0.40*  *0.143* | | **0.60**  **0.019** | | **0.81**  **0.000** | | **0.87**  **0.000** | | **0.96**  **0.000** | | **0.58**  **0.023** | |  | | **0.89**  **0.000** | | **0.84**  **0.000** | | **0.82**  **0.001** | | **0.83**  **0.001** | | **0.55**  **0.053** | | **0.86**  **0.000** | | *0.47*  *0.104* | | 0.39  0.188 | | **0.91**  **0.000** | | **0.72**  **0.005** | |  |
| **HIS** | -0.11  0.707 | | *0.50*  *0.060* | | **0.77**  **0.001** | | *0.49*  *0.062* | | **0.60**  **0.018** | | **0.74**  **0.002** | | **0.57**  **0.025** | |  | | **0.90**  **0.000** | | **0.86**  **0.000** | | **0.93**  **0.000** | | **0.64**  **0.019** | | **0.93**  **0.000** | | *0.50*  *0.081* | | 0.40  0.177 | | **0.96**  **0.000** | | **0.60**  **0.031** | |  |
| **ILE** | -0.35  0.197 | | **0.74**  **0.002** | | **0.94**  **0.000** | | **0.61**  **0.015** | | **0.85**  **0.000** | | **0.75**  **0.001** | | **0.76**  **0.001** | | **0.69**  **0.004** | |  | | **0.99**  **0.000** | | **0.85**  **0.000** | | 0.37  0.208 | | **0.75**  **0.003** | | **0.71**  **0.006** | | 0.04  0.898 | | **0.92**  **0.000** | | 0.37  .213 | |  |
| **LEU** | -0.31  0.266 | | **0.73**  **0.002** | | **0.92**  **0.000** | | **0.66**  **0.007** | | **0.89**  **0.000** | | **0.70**  **0.004** | | **0.83**  **0.000** | | **0.62**  **0.013** | | **0.97**  **0.000** | |  | | **0.78**  **0.002** | | 0.33  0.268 | | **0.68**  **0.011** | | **0.74**  **0.004** | | -0.04  0.891 | | **0.89**  **0.000** | | 0.34  0.259 | |  |
| **LYS** | 0.28  0.310 | | **0.85**  **0.000** | | *0.42*  *0.114* | | 0.34  0.208 | | 0.36  0.184 | | **0.84**  **0.000** | | 0.27  0.326 | | 0.37  0.179 | | *0.43*  *0.106* | | *0.41*  *0.127* | |  | | *0.45*  *0.120* | | **0.89**  **0.000** | | **0.57**  **0.041** | | 0.39  0.192 | | **0.91**  **0.000** | | **0.60**  **0.029** | |  |
| **MET** | 0.00  0.987 | | *0.40*  *0.139* | | **0.69**  **0.004** | | 0.31  0.261 | | *0.45*  *0.093* | | **0.70**  **0.004** | | *0.40*  *0.140* | | **0.85**  **0.000** | | **0.61**  **0.016** | | **0.52**  **0.045** | | 0.30  0.27 | |  | | **0.79**  **0.001** | | -0.09  0.771 | | **0.77**  **0.002** | | 0.52  0.068 | | **0.61**  **0.027** | |  |
| **PHE** | -0.24  .383 | | *0.45*  *0.096* | | **0.87**  **0.000** | | *0.47*  *0.078* | | **0.68**  **0.005** | | **0.68**  **0.005** | | **0.63**  **0.013** | | **0.81**  **0.000** | | **0.81**  **0.000** | | **0.77**  **0.001** | | 0.24  .380 | | **0.90**  **0.000** | |  | | **0.65**  **0.016** | | 0.06  0.847 | | **0.56**  **0.049** | | 0.28  .346 | |  |
| **SER** | 0.03  .924 | | *0.49*  *0.065* | | **0.54**  **0.037** | | **0.55**  **0.033** | | **0.51**  **0.053** | | **0.69**  **0.004** | | *0.49*  *0.061* | | **0.88**  **0.000** | | *0.50*  *0.059* | | *0.48*  *0.068* | | *0.45*  *0.096* | | **0.69**  **0.005** | | **0.58**  **0.023** | |  | | -0.34  0.251 | | **0.59**  **0.035** | | 0.16  0.592 | |  |
| **TRP** | **0.71**  **0.003** | | *0.39*  *0.148* | | -0.14  0.615 | | *-0.40*  *0.134* | | -0.30  0.278 | | 0.30  0.274 | | *-0.41*  *0.127* | | -0.05  0.871 | | -0.10  0.721 | | -0.17  0.537 | | **0.62**  **0.014** | | -0.05  0.872 | | -0.25  0.375 | | 0.01  0.964 | |  | | 0.27  0.370 | | **0.69**  **0.009** | |  |
| **TYR** | 0.32  0.249 | | **0.79**  **0.000** | | **0.67**  **0.007** | | 0.24  0.383 | | *0.47*  *0.074* | | **0.80**  **0.000** | | 0.37  0.177 | | **0.66**  **0.007** | | **0.70**  **0.004** | | **0.64**  **0.010** | | **0.60**  **0.017** | | **0.65**  **0.009** | | **0.59**  **0.020** | | **0.53**  **0.043** | | *0.47*  *0.076* | |  | | **0.56**  **0.047** | |  |
| **VAL** | -0.18  0.519 | | **0.58**  **0.024** | | **0.88**  **0.000** | | **0.58**  **0.024** | | **0.69**  **0.004** | | **0.83**  **0.000** | | **0.67**  **0.007** | | **0.91**  **0.000** | | **0.79**  **0.000** | | **0.75**  **0.001** | | **0.45**  **0.095** | | **0.89**  **0.000** | | **0.92**  **0.000** | | **0.75**  **0.001** | | -0.11  0.695 | | **0.68**  **0.005** | |  | |  |
| 1. **Pregnant Rats** | | | | | | | | | | | | | | | | | | | | | | | | | | | | | | | | | |  | |
| **SP**  **C** | | **OGDHC** | | **ALA** | | **ARG** | | **ASP** | | **GABA** | | **GLU** | | **GLY** | | **HIS** | | **ILE** | | **LEU** | | **LYS** | | **MET** | | **PHE** | | **SER** | | **TRP** | | **TYR** | | **VAL** | |
| **OGDHC** | |  | | -0.34  0.378 | | -0.25  0.511 | | **-0.67**  **.049** | | -0.21  0.584 | | *-0.58*  *0.104* | | -0.23  0.550 | | -0.32  0.394 | | -0.21  0.581 | | -0.24  0.542 | | 0.27  0.490 | | 0.24  0.530 | | -0.11  0.779 | | 0.42  0.260 | | -0.32  0.406 | | -0.32  0.398 | | .36  0.345 | |
| **ALA** | | −0.35  0.569 | |  | | **0.92**  **0.000** | | **0.75**  **0.021** | | **0.94**  **0.000** | | 0.30  0.439 | | **0.91**  **0.001** | | **0.78**  **0.014** | | **0.91**  **0.001** | | **0.93**  **0.000** | | 0.63  0.071 | | 0.49  0.183 | | **0.83**  **0.006** | | 0.39  0.296 | | *-0.58*  *0.099* | | **0.81**  **0.008** | | *0.56*  *0.114* | |
| **ARG** | | −0.33  0.582 | | 0.86  *0.063* | |  | | *0.55*  *0.125* | | **0.97**  **0.000** | | 0.02  0.968 | | **0.98**  **0.000** | | **0.77**  **0.016** | | **0.98**  **0.000** | | **0.99**  **0.000** | | **0.76**  **0.017** | | **0.69**  **0.039** | | **0.92**  **0.000** | | 0.47  0.203 | | **-0.77**  **0.015** | | **0.79**  **0.012** | | *0.64*  *0.064* | |
| **ASP** | | −0.04  0.953 | | *0.85*  *0.066* | | *0.85*  *0.067* | |  | | *0.62*  *0.076* | | **0.74**  **0.0023** | | *0.58*  *0.099* | | *0.62*  *0.075* | | 0.50  0.167 | | 0.54  0.135 | | 0.12  0.767 | | -0.05  0.897 | | 0.44  0.238 | | 0.06  0.875 | | -0.04  0.928 | | **0.66**  **0.052** | | -0.04  0.917 | |
| **GABA** | | −0.55  0.340 | | **0.88**  **0.052** | | **0.96**  0.010 | | 0.82  0.087 | |  | | 0.07  0.867 | | **0.98**  **0.000** | | **0.84**  **0.005** | | **0.97**  **0.000** | | **0.98**  **0.000** | | **0.79**  **0.012** | | **0.68**  **0.045** | | **0.95**  **0.000** | | 0.56  0.117 | | **-0.73**  **0.026** | | **0.74**  **0.022** | | **0.68**  **0.045** | |
| **GLU** | | 0.33  0.588 | | 0.49  0.398 | | 0.21  0.732 | | 0.68  0.205 | | 0.23  0.710 | |  | | -0.01  0.979 | | 0.15  0.704 | | -0.09  0.812 | | -0.03  0.934 | | -0.36  0.341 | | **-0.68**  **0.045** | | -0.18  0.640 | | -0.38  0.313 | | 0.52  0.154 | | 0.29  0.455 | | -0.51  0.160 | |
| **GLY** | | −0.51  0.380 | | 0.78  0.118 | | **0.96**  0.010 | | 0.80  0.104 | | **0.98**  0.003 | | 0.15  0.808 | |  | | **0.81**  **0.008** | | **0.98**  **0.000** | | **0.98**  **0.000** | | **0.76**  **0.017** | | **0.73**  **0.026** | | **0.94**  **0.000** | | *0.53*  *0.145* | | **-0.78**  **0.013** | | **0.75**  **0.020** | | *0.64*  *0.066* | |
| **HIS** | | *0.76*  *0.136* | | 0.24  0.700 | | 0.27  0.660 | | 0.39  0.519 | | 0.01  0.990 | | 0.34  0.576 | | 0.01  0.989 | |  | | **0.78**  **0.014** | | **0.80**  **0.010** | | 0.46  0.217 | | 0.50  0.167 | | **0.75**  **0.020** | | *0.55*  *0.123* | | -0.43  0.251 | | 0.40  0.285 | | 0.49  0.176 | |
| **ILE** | | −0.44  0.456 | | **0.93**  0.022 | | **0.98**  0.003 | | 0.85  0.071 | | **0.98**  0.003 | | 0.26  0.672 | | **0.95**  0.015 | | 0.17  0.780 | |  | | **0.99**  **0.000** | | **0.79**  **0.011** | | **0.78**  **0.013** | | **0.95**  **0.000** | | 0.50  0.169 | | **-0.80**  **0.010** | | **0.71**  **0.031** | | **0.74**  **0.023** | |
| **LEU** | | −0.42  0.479 | | **0.90**  0.036 | | **0.99**  0.001 | | 0.84  0.076 | | **0.98**  0.004 | | 0.22  0.724 | | **0.96**  0.011 | | 0.20  0.752 | | **0.99**  0.000 | |  | | **0.78**  **0.014** | | **0.74**  **0.022** | | **0.95**  **0.000** | | 0.52  0.153 | | **-0.78**  **0.014** | | **0.75**  **0.019** | | **0.71**  **0.031** | |
| **LYS** | | 0.07  0.910 | | *0.80*  *0.106* | | *0.81*  *0.095* | | 0.71  0.177 | | 0.66  0.230 | | 0.26  0.670 | | 0.61  0.275 | | 0.70  0.191 | | *0.79*  *0.112* | | *0.80*  *0.106* | |  | | 0**.80**  **0.010** | | **0.88**  **0.002** | | *0.64*  *0.061* | | **-0.84**  **0.004** | | *0.56*  *0.121* | | **0.85**  **0.003** | |
| **MET** | | −0.15  0.812 | | 0.00  0.994 | | 0.39  0.518 | | −0.08  0.893 | | 0.24  0.698 | | −0.74  0.156 | | 0.33  0.585 | | 0.21  0.740 | | 0.28  0.651 | | 0.34  0.578 | | 0.39  0.512 | |  | | **0.81**  **0.008** | | **0.67**  **0.050** | | **-0.91**  **0.001** | | 0.34  0.365 | | **0.86**  **0.003** | |
| **PHE** | | −0.04  0.951 | | 0.50  0.396 | | *0.84*  *0.077* | | 0.58  0.310 | | 0.67  0.213 | | −0.14  0.827 | | *0.74*  *0.149* | | 0.49  0.402 | | 0.72  0.166 | | *0.77*  *0.125* | | *0.78*  *0.123* | | *0.76*  *0.137* | |  | | **0.68**  **0.044** | | **-0.81**  **0.008** | | **0.71**  **0.032** | | **0.79**  **0.012** | |
| **SER** | | 0.36  0.549 | | 0.43  0.466 | | 0.60  0.288 | | 0.45  0.446 | | 0.35  0.565 | | 0.02  0.978 | | 0.36  0.546 | | 0.86  0.065 | | 0.50  0.393 | | 0.53  0.353 | | **0.89**  0.045 | | 0.61  0.272 | | 0.82  0.086 | |  | | **-0.66**  **0.052** | | 0.31  0.411 | | **0.72**  **0.030** | |
| **TRP** | | *0.85*  *0.070* | | −0.21  0.740 | | −0.45  0.444 | | 0.01  0.988 | | −0.55  0.339 | | 0.64  0.242 | | −0.59  0.295 | | 0.53  0.358 | | −0.46  0.433 | | −0.48  0.409 | | −0.08  0.903 | | −0.63  0.255 | | −0.43  0.470 | | 0.02  0.979 | |  | | *-0.57*  *0.111* | | **-0.79**  **0.011** | |
| **TYR** | | −0.08  0.900 | | *0.81*  *0.097* | | **0.96**  0.008 | | *0.86*  *0.061* | | *0.85*  *0.068* | | 0.27  0.663 | | *0.86*  *0.064* | | 0.52  0.373 | | **0.91**  0.031 | | **0.93**  0.022 | | **0.90**  0.035 | | 0.42  0.482 | | **0.89**  0.041 | | *0.77*  *0.129* | | −0.26  0.669 | |  | | 0.33  0.381 | |
| **VAL** | | *0.76*  *0.134* | | 0.27  0.662 | | 0.06  0.930 | | 0.35  0.560 | | -0.13  0.838 | | 0.63  0.257 | | -0.20  0.750 | | **0.88**  **0.051** | | 0.04  0.952 | | 0.02  0.969 | | 0.54  0.352 | | -0.25  0.683 | | 0.08  0.896 | | 0.57  0.320 | | *0.79*  *0.110* | | 0.28  0.651 | |  | |

Pearson′s correlation coefficient (upper value) and *p-*value of the correlation (lower value) are shown. Statistically significant (*p* < 0.05) positive (light grey) and negative (dark grey) correlations are marked. The lower left and upper right triangles refer to the control (C) rats (*n* = 17 in A and *n* = 13 in B) and the SP-treated (SP) rats (*n* = 8 in A and *n* = 11 in B).

**Supplementary Table 2.** Comparison of the actions of SP and acute hypobaric hypoxia on the OGDHC/amino acids network. Overall and average correlation coefficients are compared in (A), with the numbers of the positive and negative correlations compared in (B)

**A.**

| **Groups**  **Parameter** | **Non-Pregnant Rats (NP)** | | | | | | **Pregnant Rats (P)** | | | | | |
| --- | --- | --- | --- | --- | --- | --- | --- | --- | --- | --- | --- | --- |
|  | **∑** | | | **͞Х** | | | **∑** | | | **͞X** | | |
|  | **Control** | **SP** | **Hypoxia** | **Control** | **SP** | **Hypoxia** | **Control** | **SP** | **Hypoxia** | **Control** | **SP** | **Hypoxia** |
| **OGDHC** | 4,27 | 6,96 | 12,60 | 0,27 | 0,43 | 0,79 | 6,04 | 5,08 | 4,93 | 0,38 | 0,32 | 0,31 |
| **ALA** | 9,45 | 11,78 | 13,24 | 0,59 | 0,74 | 0,83 | 8,95 | 11,06 | 7,92 | 0,60 | 0,69 | 0,50 |
| **ARG** | 10,89 | 11,73 | 13,19 | 0,68 | 0,73 | 0,82 | 10,19 | 11,47 | 7,54 | 0,68 | 0,72 | 0,47 |
| **ASP** | 8,65 | 8,99 | 12,58 | 0,54 | 0,56 | 0,79 | 9,12 | 6,97 | 4,35 | 0,61 | 0,44 | 0,27 |
| **GABA** | 10,21 | 9,93 | 13,39 | 0,64 | 0,62 | 0,84 | 9,29 | 11,70 | 6,82 | 0,62 | 0,73 | 0,43 |
| **GLU** | 10,59 | 10,40 | 12,37 | 0,66 | 0,65 | 0,77 | 5,28 | 4,89 | 5,80 | 0,35 | 0,31 | 0,36 |
| **GLY** | 9,62 | 11,25 | 13,10 | 0,60 | 0,70 | 0,82 | 9,28 | 11,59 | 7,64 | 0,62 | 0,72 | 0,48 |
| **HIS** | 9,61 | 11,62 | 13,08 | 0,60 | 0,73 | 0,82 | 5,82 | 9,44 | 7,10 | 0,39 | 0,59 | 0,44 |
| **ILE** | 10,59 | 11,69 | 13,11 | 0,66 | 0,73 | 0,82 | 9,81 | 11,68 | 8,95 | 0,65 | 0,73 | 0,56 |
| **LEU** | 10,39 | 11,58 | 13,16 | 0,65 | 0,72 | 0,82 | 9,95 | 11,70 | 8,73 | 0,66 | 0,73 | 0,55 |
| **LYS** | 7,24 | 10,81 | 11,79 | 0,45 | 0,68 | 0,74 | 9,72 | 10,27 | 6,44 | 0,65 | 0,64 | 0,40 |
| **MET** | 8,42 | 6,29 | 9,02 | 0,53 | 0,39 | 0,56 | 5,67 | 9,97 | 7,80 | 0,38 | 0,62 | 0,49 |
| **PHE** | 9,89 | 7,84 | 13,00 | 0,62 | 0,49 | 0,81 | 9,21 | 11,70 | 7,65 | 0,61 | 0,73 | 0,48 |
| **SER** | 8,16 | 8,76 | 5,67 | 0,51 | 0,55 | 0,35 | 7,78 | 8,06 | 8,01 | 0,52 | 0,50 | 0,50 |
| **TRP** | 4,49 | 5,97 | 7,90 | 0,28 | 0,37 | 0,49 | 6,13 | 10,32 | 6,15 | 0,41 | 0,65 | 0,38 |
| **TYR** | 9,18 | 11,77 | 13,12 | 0,57 | 0,74 | 0,82 | 10,49 | 9,05 | 7,91 | 0,70 | 0,57 | 0,49 |
| **VAL** | 10,63 | 6,92 | 5,85 | 0,66 | 0,43 | 0,37 | 5,09 | 9,70 | 5,83 | 0,34 | 0,61 | 0,36 |
| **Sum or Average** | **152,3** | **164,3** | **196,2** | **0,56** | **0,60** | **0,72** | **137,82** | **164,67** | **119,6** | **0,54** | **0,61** | **0,44** |
| ***p* *vs* control** | *0.11* **0.007** | | | *0.12* **0.008** | | | **0.05 0.04** | | | **0.04** **0.009** | | |
| ***p SP vs Hypoxia*** | ***0.003*** | | | ***0.003*** | | | ***0.0005*** | | | ***0.0006*** | | |
| **B.** |  | | |  | | |  | | |  | | |
| **Groups**  **Parameter** | **Non-Pregnant Rats** | | | | | | **Pregnant Rats** | | | | | |
|  | **+** | | | **−** | | | **+** | | | **−** | | |
|  | **Control** | **SP** | **Hypoxia** | **Control** | **SP** | **Hypoxia** | **Control** | **SP** | **Hypoxia** | **Control** | **SP** | **Hypoxia** |
| **OGDHC** | 1 | 0 | 0 | 0 | 6 | 11 | 0 | 0 | 0 | 0 | 1 | 0 |
| **ALA** | 10 | 12 | 12 | 0 | 1 | 1 | 3 | 9 | 6 | 0 | 0 | 0 |
| **ARG** | 13 | 12 | 11 | 0 | 0 | 1 | 5 | 11 | 6 | 0 | 1 | 0 |
| **ASP** | 9 | 8 | 12 | 0 | 1 | 1 | 0 | 2 | 2 | 0 | 1 | 0 |
| **GABA** | 11 | 10 | 11 | 0 | 1 | 1 | 5 | 11 | 3 | 0 | 1 | 0 |
| **GLU** | 14 | 11 | 11 | 0 | 1 | 1 | 0 | 1 | 2 | 0 | 1 | 0 |
| **GLY** | 10 | 11 | 11 | 0 | 0 | 1 | 4 | 10 | 5 | 0 | 1 | 0 |
| **HIS** | 11 | 12 | 11 | 0 | 0 | 1 | 1 | 7 | 1 | 0 | 0 | 0 |
| **ILE** | 12 | 12 | 11 | 0 | 1 | 1 | 6 | 11 | 8 | 0 | 1 | 0 |
| **LEU** | 12 | 12 | 11 | 0 | 1 | 1 | 6 | 11 | 7 | 0 | 1 | 0 |
| **LYS** | 4 | 11 | 12 | 0 | 0 | 0 | 2 | 8 | 4 | 0 | 1 | 0 |
| **MET** | 9 | 5 | 2 | 0 | 0 | 0 | 0 | 9 | 5 | 0 | 2 | 0 |
| **PHE** | 8 | 8 | 11 | 0 | 0 | 1 | 1 | 12 | 4 | 0 | 1 | 1 |
| **SER** | 12 | 9 | 1 | 0 | 0 | 0 | 1 | 3 | 7 | 0 | 1 | 0 |
| **TRP** | 2 | 3 | 0 | 0 | 0 | 0 | 0 | 0 | 1 | 0 | 10 | 1 |
| **TYR** | 11 | 13 | 11 | 0 | 0 | 1 | 5 | 8 | 7 | 0 | 0 | 0 |
| **VAL** | 13 | 7 | 0 | 0 | 0 | 0 | 1 | 7 | 4 | 0 | 1 | 0 |
| **Sum or Average** | **162** | **156** | **138** | **0** | **12** | **22** | **40#** | **120#** | **72#** | **0** | **24** | **2** |
| ***p* *vs* control** |  | | | **0.002 0.018** | | | **0.0007 0.0009** | | | **0,0014** | | |
| ***p SP vs Hypoxia*** |  | | | ***0.03*** | | | ***0,0006*** | | | ***0.001*** | | |
| ***p NP vs P*** |  | | |  | | | **0.05 0,0009 0,0009** | | | *0.14* **0,009** | | |

The data on hypoxic rats are taken from (Graf *et al.*, 2020). For the OGDHC activity (OGDHC) and each of the amino acids, the arithmetic sum of its correlation coefficients (absolute values) to other amino acids (Σ), average correlation coefficient (͞X) calculated from the absolutre values, and total number of statistically significant positive (+) and negative (–) correlations are shown. At the bottom, the sum (in case of Σ, positive and negative correlations) or average (in case of X) of all the values in the row are shown, along with *p* values of the differences between the indicated groups, estimated by the Wilcoxon signed rank test. Non-pregnant rats: control n = 17, SP n= 13, hypoxia n=8; pregnant rats: control n = 8, SP n= 11, hypoxia n=10.

**
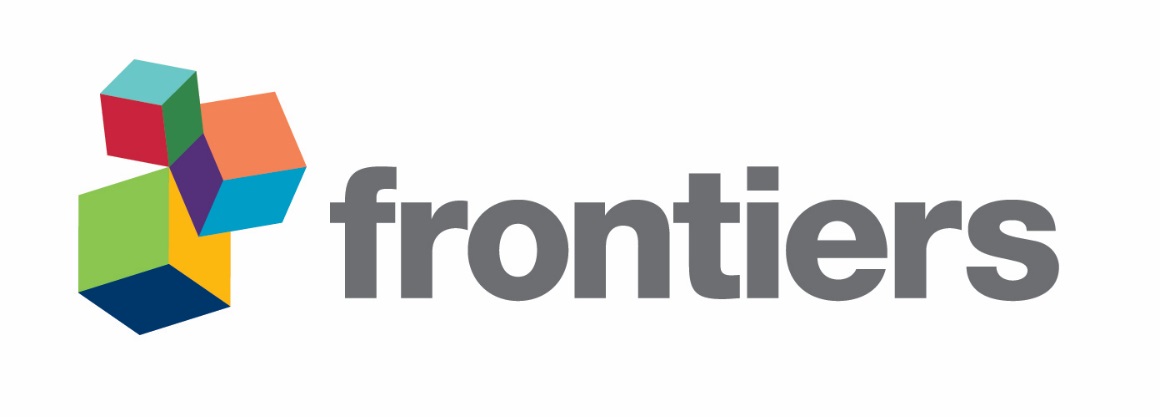
**
